# A Rare Cause of Urinary Incontinence: Vesicouterine Fistula—A Case Report

**DOI:** 10.1089/whr.2023.0121

**Published:** 2024-01-04

**Authors:** Radu Viorel Dragos, Costache Radu Cristian, Onofriescu Mircea, Morosanu Corneliu, Onofrei Pavel, Bobeica Razvan Lucian, Tanasa Vasilache Ingrid, Tanase Adina, Popescu Dan

**Affiliations:** ^1^ Department of Urology, Faculty of Medicine, University of Medicine and Pharmacy “Gr. T. Popa,” Iasi, Romania.; ^3^ Department of Obstetrics and Gynaecology, Faculty of Medicine, University of Medicine and Pharmacy “Gr. T. Popa,” Iasi, Romania.; ^2^ Department of Urology and Renal Transplantation, “C.I. Parhon” University Hospital, Iasi, Romania.; ^4^ Obstetrics and Gynaecology Department, “Cuza Voda” Obstetrics and Gynecology University Hospital, Iasi, Romania.; ^5^ Department of Morphofunctional Sciences II, Faculty of Medicine, University of Medicine and Pharmacy “Gr. T. Popa,” Iasi, Romania.; ^6^ Department of Urology, Elytis Hospital, Iasi, Romania.

**Keywords:** vesicouterine fistula, urinary incontinence, cesarean section

## Abstract

Vesicouterine fistula is a rare complication occurring mainly after cesarean sections. We present here a particular case of vesicouterine fistula (VUF) whose only symptom was urinary incontinence. We describe the diagnostic methods used and the surgical treatment used to resolve the case. A 30-year-old woman presented to the gynecology clinic with urinary incontinence that occurred 5 days postoperatively after a cesarean section. The diagnosis was made on the basis of computed tomography and cystoscopy. Treatment was surgical and consisted of excision of the fistula and suturing of the bladder and uterine wall, without interposition of the omentum. Postoperative evolution was uneventful. When the urethral catheter was removed on the 14th postoperative day, the patient presented spontaneous micturition without vaginal urine leakage. In the context of urinary incontinence after cesarean section, although it is a rare complication, we must consider the occurrence of a VUF.

## Introduction

Cesarean section is a common procedure in our region, and often a large number of women have two or more cesarean sections in their lifetime, which is associated with an increased risk of surgical complications.^1^ One of these complications is vesicouterine fistula (VUF), which occurs in 1%–9% of all urogenital fistulas.^2–4^ VUF is an abnormal communication between the bladder and uterus and can have a variety of causes, including surgical, obstetrical, radiation necrosis, or tumor disease.^5^

Because of the rarity of this complication, there are few reports in the literature regarding this entity, which presents with a variety of symptoms and different diagnostic methods. Most patients present with a triad of symptoms known as Youseff syndrome and are usually diagnosed by hysterography.^6–8^ In 83%–93% of all cases, iatrogenic injury during lower segment cesarean section is the main cause of VUF. Symptoms may occur immediately after cesarean section, later after surgery, or even months later.^9^

We present a particular case with a VUF that occurred after the second cesarean section presenting as single symptom urinary incontinence. We describe the diagnostic methods and the treatment we used to cure the patient.

### Case Presentation

**FIG. 1.**
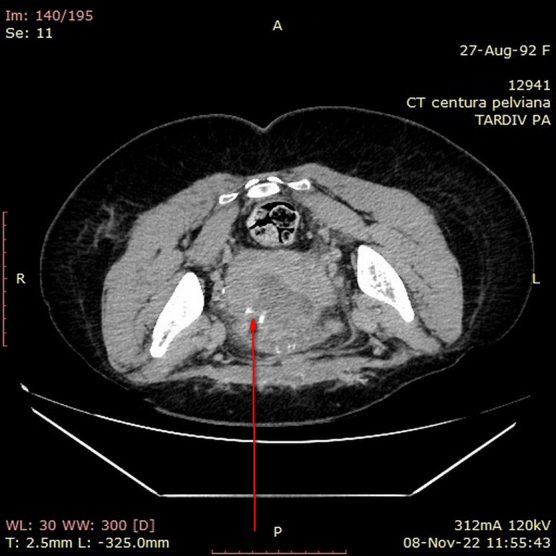
Contrast passing through the two cavities (CT, axial plane). CT, computed tomography.

**FIG. 2.**
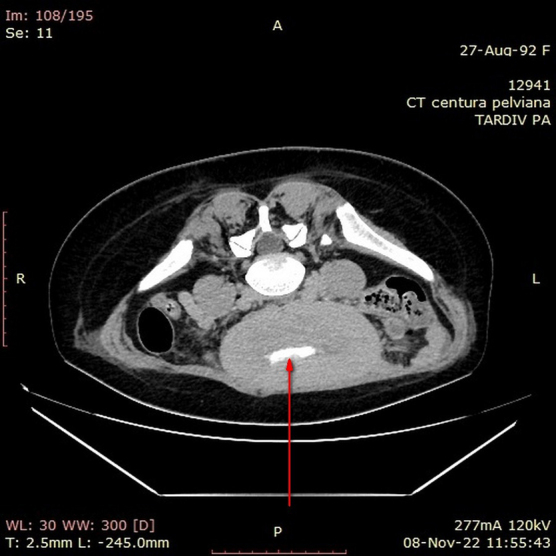
Contrast in the uterine cavity (CT, axial plane).

A 30-year-old female, with good socioeconomic status, 167 cm tall, and weighing 65 kg, underwent cesarean section surgery 3 weeks ago in another gynecological service. Five days after surgery, the patient noted vaginal fluid leakage with a decrease in urine volume, so a Foley urethral catheter was inserted, after which the vaginal fluid leakage stopped. Abdominal–pelvic computed tomography (CT) with contrast was performed, which showed the presence of a communication between the bladder and the uterus, with contrast passing from the bladder to the uterus and vagina (Figs. 1 and 2). For this reason, she was referred to the gynecology clinic at Cuza-Vodă University Hospital.

The patient's medical history indicates that she had a cesarean section 3 years ago, which was without complications. On vaginal examination with valves, the cervix was without lesions, physiological leucorrhea was present. On digital vaginal examination, the cervix was of normal consistency and the uterine body was of normal size.

Laboratory tests were normal, and uroculture was negative (the patient underwent levofloxacin treatment during the first week after Foley catheter insertion). To confirm the suspected diagnosis, a cystoscopic examination was performed, 3 weeks after the cesarean section, which revealed an ulcerated and inflamed area on the posterosuperior wall at a considerable distance from the ureteral orifices. The affected area was ∼2 × 2 cm and had a perilesional zone of inflammation 1 cm thick around the lesion. Once the bladder was filled with saline solution, a fluid vaginal leakage occurred, confirming communication between the bladder and the genital tract.

**FIG. 3.**
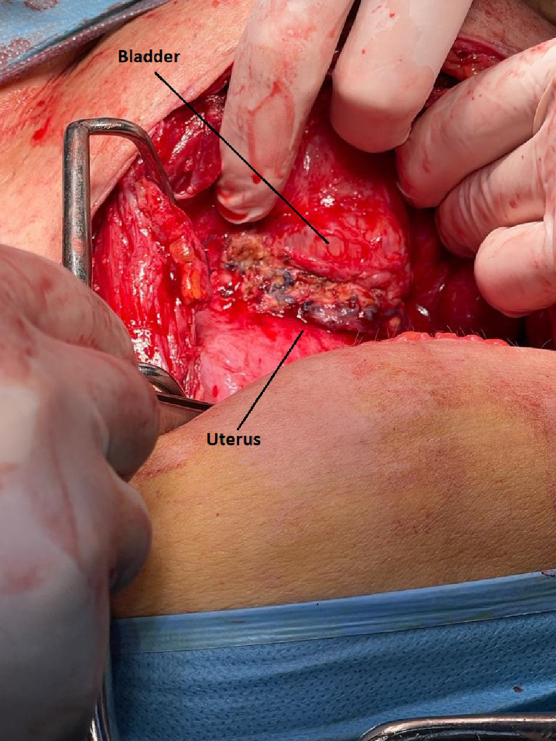
Vycril sutures on the uterus, passed also through the bladder wall.

Because the fistula persisted 2 weeks after insertion of the Foley urethral catheter, it was decided to treat the VUF surgically. Twenty-four days after cesarean section, a mixed team of gynecologists and urologists performed the operation. After a Pfannenstiel incision and entering the peritoneal cavity, an inflamed parietal and visceral peritoneum, a normal-sized uterus (24 days postpartum), a normal appearance of the ovaries on both sides, a normal appearance of the fallopian tubes with tubal ligations on both sides, an ascending urinary bladder adherent to the anterior surface of the uterus (Fig. 3), on the median line, with inflammatory edema at this level and with multiple Vycril sutures on the uterus, which were passed also through the bladder wall (Fig. 3), were revealed.

**FIG. 4.**
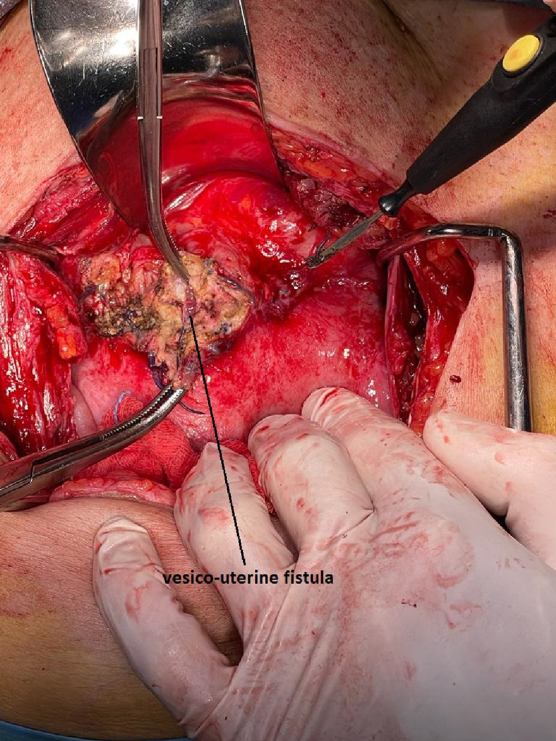
The vesicouterine fistula.

**FIG. 5.**
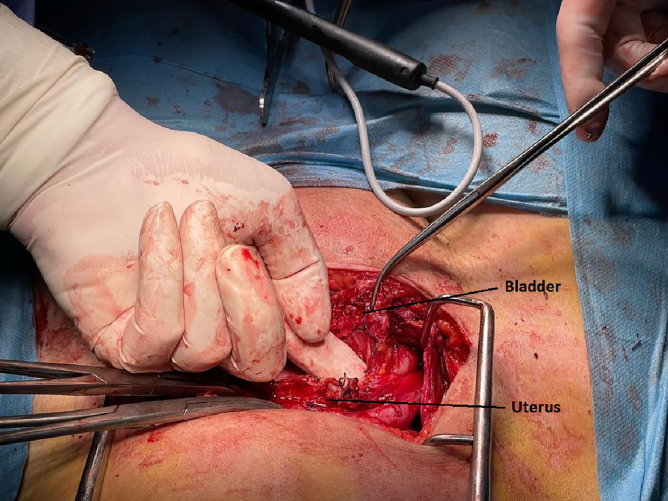
Separation of the bladder wall from the uterine wall.

Dissection of the posterosuperior bladder wall and anterior uterine wall was performed, highlighting the fistula (Fig. 4), followed by separation of the bladder wall from the uterine wall (Fig. 5), its excision along with the inflamed margins of the fistula at both the bladder and uterine levels. Once the bladder was opened to a length of ∼7 cm, the integrity of the ureteral orifices was verified, being at a safe distance from the cystotomy; therefore, no Double J ureteral stents were placed intraoperatively.

**FIG. 6.**
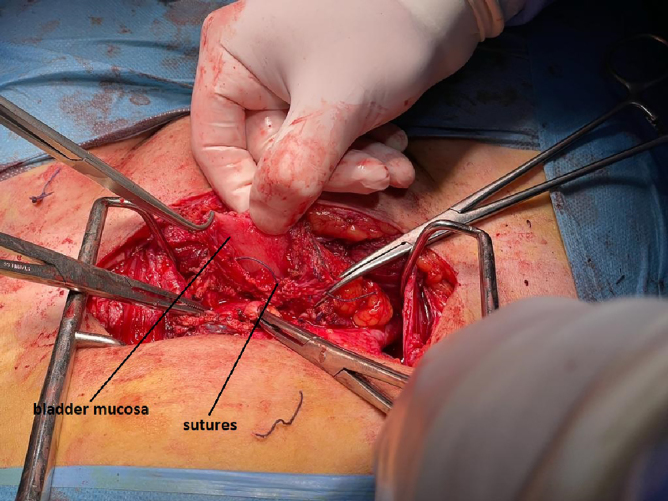
Absorbable sutures objectified passing through the uterine and bladder walls.

At the level of the fistula, numerous absorbable sutures (Vycril) were objectified, which passed through the uterine and bladder walls (Fig. 6), and were extracted. Cystorrhaphy was performed in two planes with Vycril, and the filling of the bladder with methylene blue was used to check the tightness of the suture. The hysterorrhaphy was performed using separate sutures of Vycril. Because the bladder and uterine tissues were well vascularized and the sutures were watertight, we did not consider necessary to use the omentum interposition and did not use a fibrin sealant patch in place.

A drainage tube was placed in the Douglas space before closing the abdominal wall. Total operative time was 120 minutes, with blood loss of <100 mL. The postoperative evolution was uneventful, the drain tube was removed on postoperative day 2, and the patient was discharged with a Foley urethral catheter inserted. Two weeks postoperatively, the Foley catheter was removed, and the patient presented spontaneous micturition with clear urine, without vaginal fluid loss. Control ultrasonography of the urinary system revealed the absence of pyelocaliceal distension and normal parietal aspect of the bladder, which was examined at full capacity.

We did not perform retrograde cystography to check the integrity of the bladder because we believe that 2 weeks is sufficient to ensure the healing of the bladder wall. Postmicturition bladder residue was absent. The patient returned for follow-up 3 months after surgery with no urinary incontinence and a normal menstrual cycle. After that, we did not consider follow-up necessary, since in our experience, there was no risk of recurrent fistulization. The patient should be carefully monitored in case of further pregnancy.

## Discussions and Conclusions

The clinical case we presented is intended to contribute to a better understanding of this rare disease, whose clinical presentation is as varied as the methods of diagnosis and treatment. Because it is a rare disease, most cases presented in the literature are case reports or case series.^3,7,9,10^ Most fistulas occur after multiple cesarean sections, usually after three or more,^8,11–13^ but they can also occur after the second cesarean section,^14,15^ as well as other urological complications.^1^ This complication usually occurs after a lower cesarean section,^10,16^ and it is due to inadequate reflection of the bladder wall by the lower uterine segment.^17^

Some authors have described a symptomatic triad first reported by Youseff,^6^ the triad that bears his name, consisting of amenorrhea, cyclic hematuria, and urinary incontinence. However, just as in the case we presented, patients may present with urinary incontinence only,^12,14^ suggesting that urine passes from the bladder into the uterus but not uterine contents into the bladder. This has been reported in patients diagnosed months after cesarean section.^8,11^ The hematuria that occurs immediately after surgery is the result of bladder injury and not the passage of uterine contents into the bladder.^13^

Some authors consider that the method of choice in the diagnosis of VUF is hysterography and hysteroscopy,^7,8^ which are better than urography or contrast CT because of the high pressure required in the urinary bladder for the contrast to pass into the uterus. However, other authors established the diagnosis using contrast CT^12,14^ as in our case. At least two imaging investigations are recommended for a correct diagnosis, most commonly cystoscopy followed by CT.^8,14^

As for treatment, a few authors have reported good results with conservative treatment such as Foley urethral catheter, fulguration, and hormonal therapy,^4,18^ whereas most presented surgical resolution of the fistula^11,12,14,19^ with very good postoperative results. The surgical treatment consists of excision of the fistula and also of all previous uterine sutures caught in the bladder wall, followed by restoration of the uterine and bladder walls by suturing with slowly absorbable sutures. The case we reported has several peculiarities. The patient was quickly diagnosed as a postcesarean section patient with urinary incontinence after CT with contrast.

Moreover, the surgical procedure to correct the fistula was performed in the first postoperative month and did not wait until 3 months postoperatively to maximally reduce inflammation, thus shortening the patient's suffering and her social reintegration, improving in that way the quality of life and diminishing the associated psychological burden. We believe that in the absence of urinary or genital infection, which may compromise the resolution of the fistula, early intervention is possible, of which our case is an example. As an alternative to open surgery, laparoscopic and laparoendoscopic single-site surgery (LESS) procedures have been described in the literature, which have the advantage of causing less postoperative pain, requiring fewer analgesics, and allowing a faster recovery.^20^

However, in some cases, due to the numerous adhesions and multiple inflammatory processes, the procedure cannot be performed in this manner, and conversion to open surgery is required. To avoid these disadvantages, as well as the necessity of the large opening of the bladder wall that increases the morbidity of the procedure, Przudzik et al. proposed and reported the first transvesical laparoendoscopic single-port surgery (T-LESS) repair,^21^ which can be a promising treatment solution for iatrogenic VUF in experienced hands, but further studies on higher number of patients must be performed in the future to determine the feasibility of this approach.

In conclusion, in cases of urinary incontinence after multiple cesarean sections, a VUF must be considered in the differential diagnosis. In this case, early surgical intervention can be a choice and it can shorten the patient's suffering.

## Institutional Review Board Statement

The study was conducted in accordance with the Declaration of Helsinki, and approved by the ethics committee of “Cuza-Voda” University Hospital, Iasi, Romania (protocol code 115, February 2023).

## Informed Consent Statement

Written informed consent was obtained from the patient for publication of this case report and accompanying images.

## Abbreviations Used

CT: computed tomography
VUF: vesicouterine fistula

## References

[1] Radu VD, Pristavu AI, Vinturache A, et al. Risk factors for urological complications associated with caesarean section—A case-control study. Medicina (Kaunas) 2022;58(1):123; doi: 10.3390/medicina58010123 35056431 PMC8779572

[2] Iloabachie GC, Njoku O. Vesico-uterine fistula. Br J Urol 1985;57(4):438–439. doi: 10.1111/j 4027516

[3] Hadzi-Djokic JB, Pejcic TP, Colovic VC. Vesico-uterine fistula: Report of 14 cases. BJU Int 2007;100(6):1361–1363; doi: 10.1111/j.1464-410X.2007.07067.x 17590179

[4] Milani R, Cola A, Frigerio M, et al. Repair of a vesicouterine fistula following cesarean section. Int Urogynecol J 2018;29(2):309–311; doi: 10.1007/s00192-017-3506-1 29147755

[5] Porcaro AB, Zicari M, Zecchini Antoniolli S, et al. Vesicouterine fistulas following cesarean section: Report on a case, review and update of the literature. Int Urol Nephrol 2002;34(3):335–344; doi: 10.1023/a:1024443822378 12899224

[6] Rao MP, Dwivedi US, Datta B, et al. Post caesarean vesicouterine fistulae—Youssef syndrome: Our experience and review of published work. ANZ J Surg 2006;76(4):243–245; doi: 10.1111/j.1445-2197.2006.03591.x 16681542

[7] Smayra T, Ghossain MA, Buy JN, et al. Vesicouterine fistulas: Imaging findings in three cases. AJR Am J Roentgenol 2005;184(1):139–142; doi: 10.2214/ajr.184.1.01840139 15615964

[8] Sersam LW, Al-Azzawi IS, Findakly SB. Uterovesical fistula as an uncommon complication following cesarean delivery: A case report. J Obstet Gynaecol India 2022;72(Suppl 2):389–391; doi: 10.1007/s13224-022-01656-5 PMC9701284 36457424

[9] el Fassi MJ, Tazi K, Karmouni T, et al. Le syndrome de Youssef (fistule vésico-utérine): À propos de trois observations [Youssef syndrome (vesico-uterine fistula): Three case reports]. Ann Urol (Paris) 2003;37(4):184–186; doi: 10.1016/s0003-4401(03)00052-4 12951710

[10] Rajamaheswari N, Chhikara AB. Vesicouterine fistulae: Our experience of 17 cases and literature review. Int Urogynecol J 2013;24(2):275–279; doi: 10.1007/s00192-012-1798-8 22592760

[11] Bağbancı MŞ, Emir ML, Dadalı M, et al. Vesicouterine fistula, a rare cause of genitourinary fistula. Turk J Urol 2014;40(4):251–254; doi: 10.5152/tud.2014.70846 26328188 PMC4548370

[12] Shafqat G, Khan A, Azam S, et al. Multipara with utero-vesical fistula following repeat cesarean section: A rare iatrogenic complication. Radiol Case Rep 2021;16(12):3940–3944; doi: 10.1016/j.radcr.2021.09.048 34712373 PMC8529200

[13] Katirci Y, Özdemir AZ, Güven D, et al. Uterovesical fistula after uterine compression suture. J Exp Clin Med 2022;39(1)298–299;

[14] Symeonidis EN, Sdralis E, Symeonidis A, et al. Vesicouterine fistula (VUF) as a rare urogenital complication managed with delayed surgical repair: A case report and review of the literature. Case Rep Obstet Gynecol 2018;2018:2394896; doi: 10.1155/2018/2394896 30473897 PMC6220400

[15] Bettez M, Breault G, Carr L, et al. Early versus delayed repair of vesicouterine fistula. Can Urol Assoc J 2011;5(4):E52–E55; doi: 10.5489/cuaj.10065 21806894 PMC3148396

[16] Tancer ML. Vesicouterine fistula—A review. Obstet Gynecol Surv 1986;41(12):743–753; doi: 10.1097/00006254-198612000-00001 3540759

[17] Talla P, Ekotomati M, Brünisholz Y, et al. Consider the risk of vesicouterine fistula in the event of intermittent fluid vaginal discharge after a cesarean section. Front Surg 2017;4:58; doi: 10.3389/fsurg.2017.00058 29090213 PMC5650966

[18] Murtaza B, Niaz WA, Mahmood A, et al. Vesicouterine fistula. J Coll Physicians Surg Pak 2014;24 Suppl 2:S86–S88. 24906282

[19] Shelbaia AM, Hashish NM. Limited experience in early management of genitourinary tract fistulas. Urology 2007;69(3):572–574; doi: 10.1016/j.urology.2007.01.058 17382171

[20] Symeonidis EN, Nasioudis D, Economopoulos KP. Laparoendoscopic single-site surgery (LESS) for major urological procedures in the pediatric population: A systematic review. Int J Surg 2016;29:53–61. 27000720 10.1016/j.ijsu.2016.03.040

[21] Przudzik M, Borowik M, Łesiów M, et al. Laparoendoscopic single port transvesical repair of an iatrogenic vesicouterine fistula. A three-year follow-up. Cent European J Urol 2020;73(3):387–388. 10.5173/ceju.2020.0169 PMC7587495 33133672

